# When fever is not malaria in Latin America: a systematic review

**DOI:** 10.1186/s12916-020-01746-z

**Published:** 2020-09-21

**Authors:** José Moreira, Janaina Barros, Oscar Lapouble, Marcus V. G. Lacerda, Ingrid Felger, Patricia Brasil, Sabine Dittrich, Andre M. Siqueira

**Affiliations:** 1 Laboratório de Pesquisa Clínica em Doenças Febris Agudas, Instituto Nacional de Infectologia Evandro Chagas, Fundacao Oswaldo Cruz, Rio de Janeiro, Brazil; 2 Programa de Pós-Graduação em Pesquisa Clínica em Doenças Infecciosas, Instituto Nacional de Infectologia Evandro Chagas, Fundacao Oswaldo Cruz, Rio de Janeiro, Brazil; 3 Pan-American Health Organization Office in Suriname, Paramaribo, Suriname; 4 Programa de Pós-Graduação em Medicina Tropical, Universidade do Estado do Amazonas, Manaus, Brazil; 5 Instituto de Pesquisa Clínica Carlos Borborema, Fundacao de Medicina Tropical Dr Heitor Vieira Dourado, Manaus, Brazil; 6 Instituto Leonidas e Maria Deane, Fundacao Oswaldo Cruz, Manaus, Brazil; 7 Swiss Tropical and Public Health Institute, Basel, Switzerland; 8 Foundation for Innovative New Diagnostics, Geneva, Switzerland

**Keywords:** Non-malaria febrile illness, Malaria, Latin America, Fever, Public health

## Abstract

**Background:**

In malaria-endemic countries, febrile episodes caused by diseases other than malaria are a growing concern. However, limited knowledge of the prevalent etiologic agents and their geographic distributions restrict the ability of health services to address non-malarial morbidity and mortality through effective case management. Here, we review the etiology of fever in Latin America (LA) between 1980 and 2015 and map significant pathogens commonly implicated in febrile infectious diseases.

**Methods:**

A literature search was conducted, without language restrictions, in three distinct databases in order to identify fever etiology studies that report laboratory-confirmed fever-causing pathogens that were isolated from usually sterile body sites. Data analyses and mapping was conducted with Tableau Desktop (version 2018.2.3).

**Results:**

Inclusion criteria were met by 625 publications corresponding to data relative to 34 countries. Studies using serology (*n* = 339) predominated for viral infections, culture (*n* = 131) for bacteria, and microscopy (*n* = 62) for fungi and parasites. The pathogen groups most frequently reported were viral infections (*n* = 277), bacterial infections (*n* = 265), parasitic infections (*n* = 59), fungal infections (*n* = 47), and more than one pathogen group (*n* = 24). The most frequently reported virus was dengue virus (*n* = 171), followed by other arboviruses (*n* = 55), and hantavirus (*n* = 18). For bacteria, *Staphylococcus* spp. (*n* = 82), *Rickettsia* spp. (*n* = 70), and *Leptospira* spp. (*n* = 55) were frequently reported. Areas with biggest gaps on etiology of fever were apparent.

**Conclusions:**

This review provides a landscape of pathogens causing febrile illness other than malaria in LA for over 30 years. Our findings highlight the need to standardize protocols and report guidelines for fever etiology studies for better comparability of results and improved interpretation. Lastly, we should improve existing national laboratory surveillance systems, especially from low- to middle-income countries, to inform global fever policy priorities and timely identify emerging infections threats.

**Study registration:**

PROSPERO systematic review registration number: CRD42016049281

## Background

Malaria—the disease caused by the *Plasmodium* parasite—has been for so long responsible for a significant proportion of fever in the Americas, especially within the Amazon basin. Due to its declining incidence in recent periods, clinicians faced several diagnostic and treatment dilemmas when patients present with fever other than malaria [1–3]. Current guidelines are hard to interpret and results in the febrile illness being left untreated or treated with inappropriate antimicrobials on the one hand, and over-treatment of self-limiting conditions with antimicrobials on the other hand. This practice has important implications for the development of antimicrobial resistance (AMR) [4, 5].

The differential diagnosis of non-malaria fever illness (NMFI) is vast. It can present a challenge to frontline healthcare workers in Latin America (LA). Additional complexities in resource-poor settings resulted in higher morbidity and mortality, wasted resources, and accelerated AMR. Many fever illnesses share the signs and symptoms hampering a clinical diagnosis, and even when sophisticated diagnostic tests are available (i.e., in a research setting), in almost half of the cases, the microbiological cause of the illness remains obscure. The “true” infection can be challenging to differentiate from colonization, and illness can be attributed to more than one pathogen. This challenge is compounded by the lack of appropriate diagnostic technologies at the district level, where the majority of affected patients seek care. Finally, to further aggravate the diagnosis and management of fever illness, limited clinical expertise does exist for identifying a severe illness in the community setting, as doctors are usually found at higher levels.

Understanding the significant causes of NMFI is crucial. Epidemiological surveillance data collected routinely on a regional basis is a good source but often not readily available. In the absence of these, etiological fever studies have been conducted in LA and provided some indication of the clinical spectrum of febrile illness in malaria-endemic areas. However, they tend to be conducted in large reference centers hence tricky to generalize, with a focus on specific clinical syndromes (i.e., fever without focus), and the studies also lack standardization in methods and laboratory approaches [2]. Despite these shortcomings, such data should be galvanized in order to encourage optimal use of information that is already available [6].

Here, we undertake a systematic review of the literature searching for the known records of pathogens causing a febrile illness in LA between 1980 and 2015. This review aims to provide a more reliable assessment about the geographical distribution of febrile illness over time, identify gaps in knowledge, and draw up a research agenda that reflects priorities in the identification the etiologies of fever in children and adults living in malaria-endemic areas.

## Methods

### Protocol and registration

The study protocol for this systematic review was registered at the PROSPERO database (CRD42016049281).

### Search strategy

This systematic collection of data followed the Preferred Reporting Items for Systematic reviews and Meta-Analyses (PRISMA) guidelines to identify published articles describing NMFI in LA [7]. A literature search of three databases (Ovid Medline, Latin-American health databases, and Embase) was performed from 1 January 1980 until 4 August 2015, without language restriction. Supplementary File describes the search strategy applied in each database, including keywords and specific search terms for diseases and countries of the LA region. The search was not limited by study design or patient age but was restricted to articles published between 1980 and 2015. The resulting catalog of references was subsequently managed with Mendeley.

### Eligibility

Comprehensive inclusion and exclusion criteria were predefined to facilitate the objective screening of papers. Studies were eligible if they described the laboratory detection of pathogen causing febrile illness in LA (rather than clinical definition alone) as well as studies that reported pathogens isolated from a normally sterile body sites (i.e., blood, bone marrow, cerebrospinal fluid), studies that enrolled either outpatient or inpatients, and studies that clearly stated the total number of individuals tested and those that were positive for infection. Although urine is considered a potentially sterile site, we excluded studies that isolated organisms in this medium as contamination is frequently seen if appropriate countermeasures are not in place.

We excluded the following studies: (i) publications with primarily focus on malaria, HIV, or tuberculosis; (ii) non-clinical investigations; (iii) drug or vaccine trials; (iv) studies conducted in travelers returning to their home countries outside LA; and (v) reports describing nosocomial infections. We excluded studies that focused primarily on HIV or tuberculosis populations because we could potentially select pathogens that are most often associated with immunodepression (i.e., *Pneumocystis jirovecii*, *Cryptococcus neoformans*, JC virus).

### Study selection

Title and abstracts of the retrieved studies using the search strategy were screened independently by two reviewers to identify studies potentially eligible for inclusion. The full text of the potentially eligible studies were retrieved and independently assessed for eligibility by two authors. All conflicts of opinion and uncertainties were discussed and resolved by consensus with third-party reviewers. Attempts were also made to clarify with the corresponding authors regarding any uncertainties or missing data in selected reports.

### Data extraction

An online database, hosted by the Infectious Diseases Data Observatory (IDDO), was developed to capture variables of interest for this review (http://demo.wwarn.org:8080/NMFIDataManager/#/). Full text review was performed for all the selected papers, and data were extracted into a standardized form that includes the following variables: first author, year of publication, study start year, study end year, study design, age range, number of patients tested for each infection and those confirmed positive, laboratory method used to identify the pathogen, specimen type, and geo-coded study sites parameters.

### Risk of bias assessment

The risk of bias assessment was not done given the heterogeneity of the studies included.

### Strategy for data synthesis

Descriptive statistics were used to characterize categorical and numerical variables, frequencies and percentages for categorical variables and mean/median for continuous one. The heterogeneity in the methods used for the selected studies prevented a meta-analysis between the studies being performed.

The unit of analysis was the number of articles and not the patients. As some articles identified more than one pathogen, the number of pathogens exceeds the total number of articles included.

Data analysis was performed in the Tableau Desktop for Mac (version 2018.2.3, Tableau Software, Inc., 2018).

### Definitions

For this review, LA was defined as a group of countries or territories that are situated south of the USA, thus including the Caribbean.

Non-malaria febrile illness is defined as fever caused by diseases other than malaria.

Fever series refers to studies with a precise number of individuals tested and those who tested positive. Case series refers to individual case reports or case series describing the presence of a specific febrile illness in a particular place at a particular time.

Outbreak investigation refers to studies reporting an outbreak of a specific febrile illness in a targeted geographical region.

Seroprevalence studies refer to studies investigating a particular febrile illness in symptomatic or asymptomatic subjects during a specific time point.

We categorized patients’ age groups as follows: neonate’s studies (< 30 days of life), infants (< 12 months), children (1–12 years), adult (≥ 13 years), and all age groups (pediatric and adult population). The unspecified age category was referred to when a study did not mention any information regarding age.

### Data visualization and online interactive map

The etiological maps were done with Tableau Desktop for Mac (version 2018.2.3, Tableau Software, Inc., 2018). Each study site was geo-coded to find its latitude and longitude using Google maps.

Moreover, a map derived by our data (using this systematic review) was built and hosted on the WWARN web site (http://www.wwarn.org/surveyor/NMFIv3/#2). The Non-Malaria Febrile Illness Map is an interactive platform that shows the geographical location of an organism causing fever in malaria-endemic regions since 1980. A detailed search could be specified according to a country of origin, underlying pathogen, year, or age group. Each study is mapped with primary descriptive data, including study type and frequency of a positive result that can be obtained by clicking the marker on the interactive map.

## Results

### Overview

A total of 23,023 publications were identified from databases, and 625 (2.71%) were included after full-text review corresponding to data from 34 countries. Fig. S1 shows the flowchart of the included studies. Studies were conducted between 1944 and 2015. *N* refers to the number of publications included. Sixty percent (*n* = 378) were published in English, and the others in Spanish, Portuguese, and French. The number of papers published each year that the met inclusion criteria substantially increased between 1981 and 2015 (Fig. S2).

### Spatial distribution

**Fig. 1:**
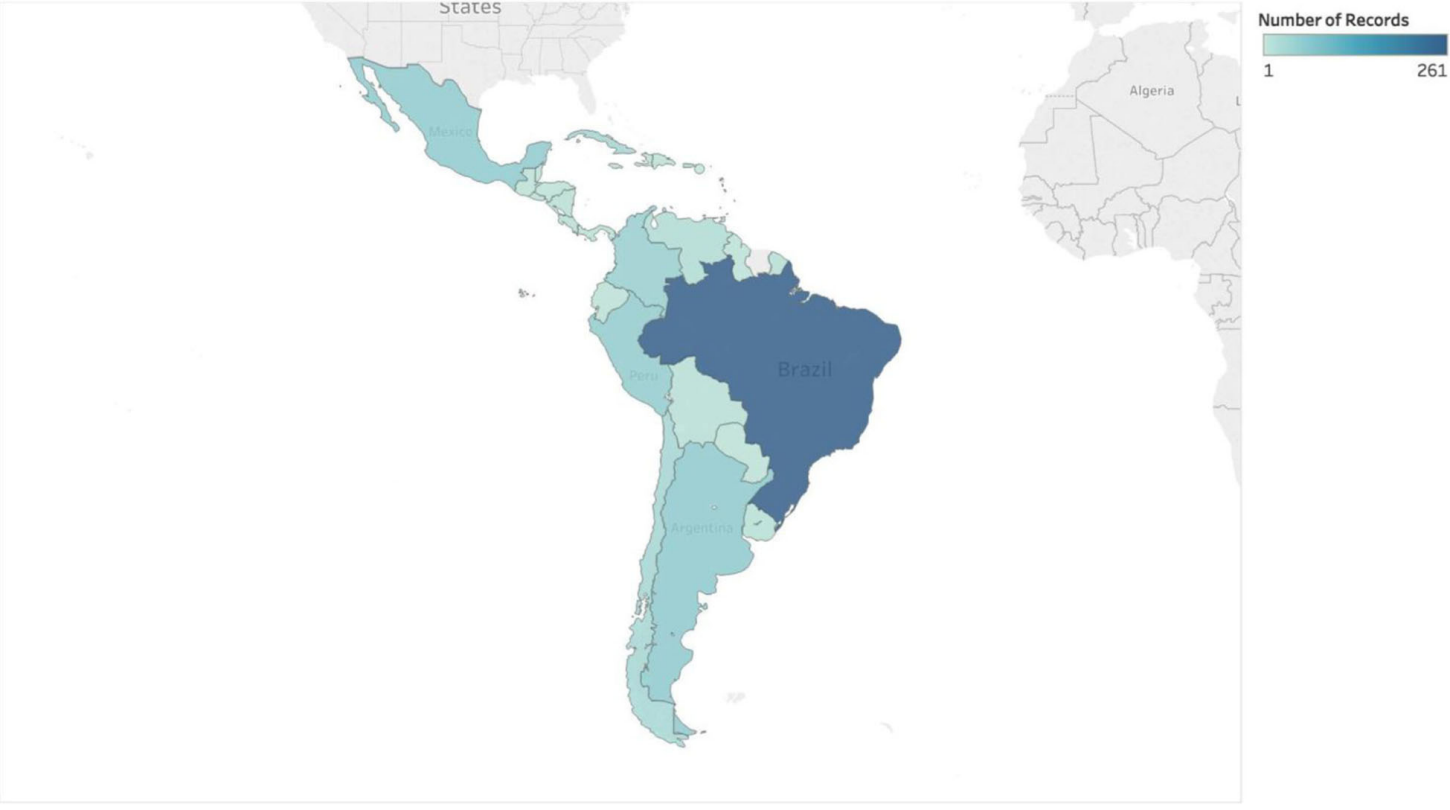
Geographical distribution and number of selected studies published between 1980 and 2015 in the different Latin America countries. Geographical distribution and number of studies included in the review by country between 1980 and 2015

The geographical distribution of the studies included was heterogeneous (Fig. 1). The majority of the reports were from South America (76.8%), followed by Caribbean (10.8%), North America (8.16%), and Central America (4.16%). The countries contributing the most reports to this systematic review were Brazil (261, 41.7%), Argentina (53, 8.4%), Mexico (51, 8.1%), and Peru (49, 7.8%). Fig. S3 depicts the location of each study site. The central and Midwestern parts of Brazil, as well as the South and Southeast of Argentina and Chile, did not have any study sites.

### Descriptive summary of study type

The mean study length was 1.63 [range 0–41] years. More than half of the publications consisted of case series (340, 54.4%), while 122 (19.5%) were fever series, and 146 (23.36%) had other designs. In seventeen reports, study design could not be ascertained. Fig. S4 shows the distribution of the main study types per country.

### Study population

Adults were studied in 229 (36.6%) reports, followed by children in 70 (11.2%), infants in 9 (1.44%), and neonates in 6 (0.96%). A total of 215 (34.4%) reports included patients of all age groups, while age was not specified in 96 (15.36%). Studies described medical ward or intensive care admission in 322 (51.5%), and in 192 (30.7%), a history of febrile illness was reported.

### Sample sources

Blood was the specimen analyzed most often (519, 83.04%). Cerebrospinal spinal fluid was collected in 35 (5.6%), bone marrow in 19 (3.04%), joint aspirates in 3 (0.48%), and multiple sample sources in 49 (7.84%) articles. Blood specimens were the dominant medium to diagnose pathogens throughout time (Fig. S5).

### Laboratory methods

**Fig. 2:**
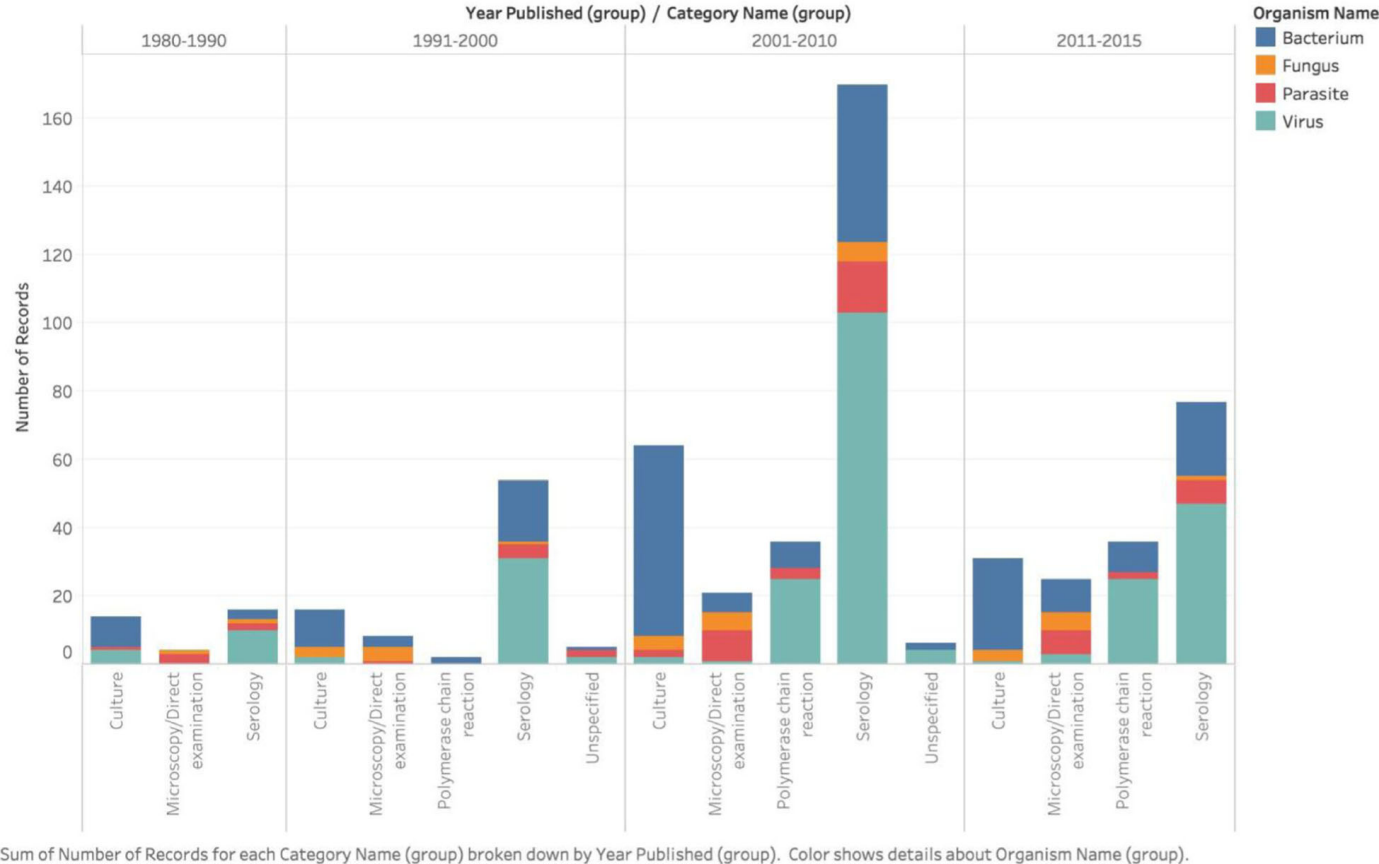
Laboratory methods according to pathogen over time, 1980–2015. Laboratory methods described in the included articles by organism category and time frame

Serology was predominantly used for identifying viral infections, culture methods for bacteria, and microscopy/direct examination for fungi and parasites. A recent surge was seen in the use of molecular methods since 1990. Trends over time in the use of laboratory techniques for given pathogens are presented in Fig. 2.

### Etiology of non-malarial febrile illness in Latin America and etiology maps

The etiologies of NMFI most frequently reported were viral infections (*n* = 277), bacterial infections (*n* = 265), parasitic infections (*n* = 59), fungal infections (*n* = 47), and more than one pathogen group (*n* = 24). Of the 24 articles reporting multiple groups, 12 reported bacteria and viruses, six reported bacteria and fungi, three reported bacteria and parasites, two bacteria, viruses and parasites, and one reported bacteria, fungi, and parasites.

**Fig. 3:**
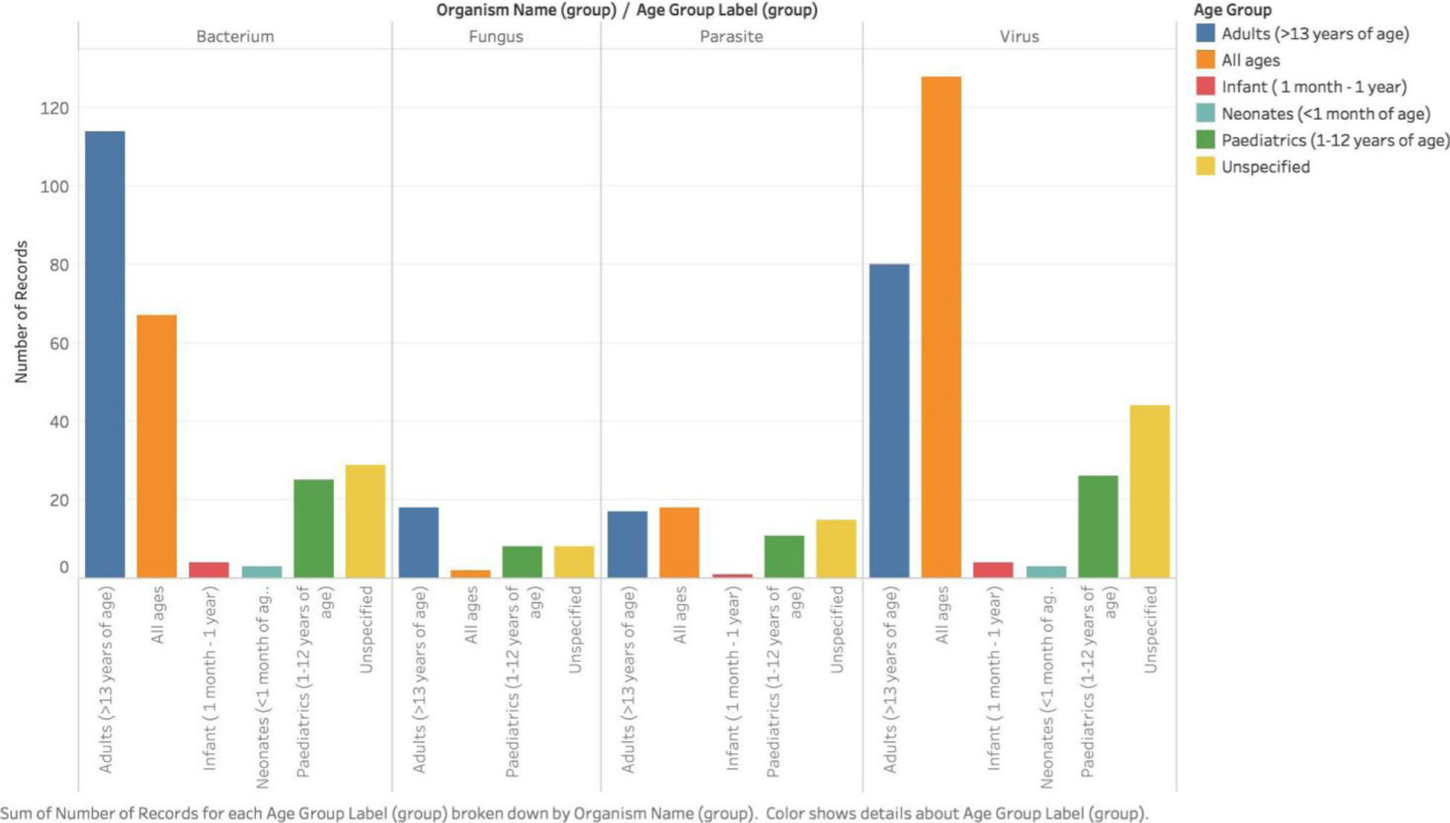
Etiology of non-malaria febrile illness according to age groups, 1980–2015. Etiology of non-malaria febrile illness in Latin America according to age group. Note that in some reports, age was not mentioned, and we referred to these studies as “unspecified age group”

The etiology of pathogen groups, according to age, is shown in Fig. 3. Fig. S6 shows the distribution of the main pathogen groups in LA.

### Viral infections

In all age groups, the most reported viral infections were dengue virus (DENV) (*n* = 171), other arthropod-borne viruses (*n* = 55), hantavirus (*n* = 18), human herpesviruses (*n* = 18), parvoviruses (*n* = 11), and respiratory borne-viruses (*n* = 11). Similarly, among adults, the viruses most reported were DENV (*n* = 47), other arthropod-borne viruses (*n* = 22), hantavirus (*n* = 11), and human herpesvirus (*n* = 4).

### Dengue

**Fig. 4:**
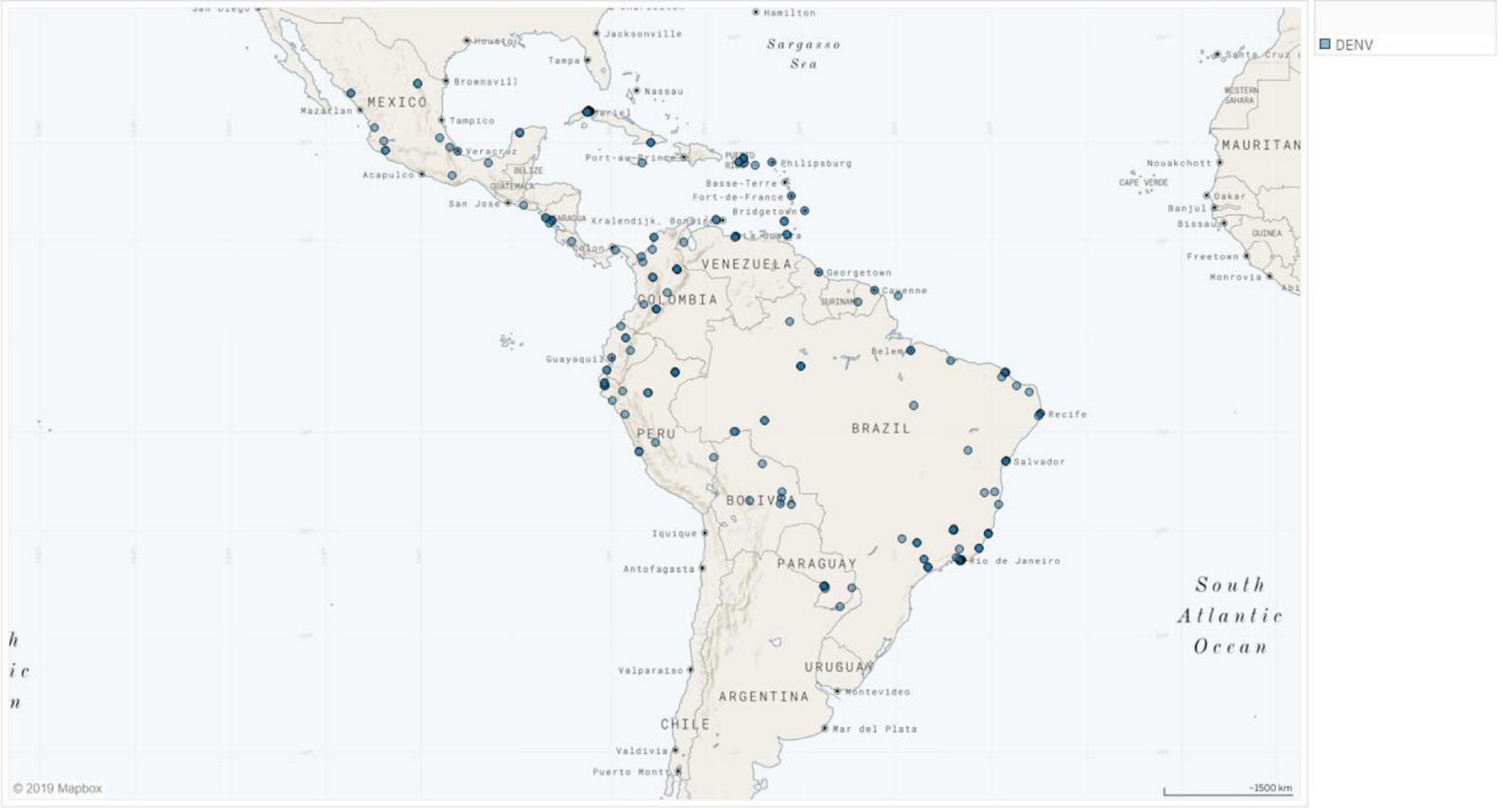
Dengue in Latin America, 1980–2015. Geographical distribution of study sites where dengue has been reported in Latin America. DENV denotes dengue virus

Study sites where DENV has been reported are shown in Fig. 4. DENV were predominantly reported in studies with adult patients (Fig. S7). The serotypes most frequently reported were DENV-2, followed by DENV-1, DENV-3, and DENV-4 (Fig. S8). All serotypes increase over time apart from DENV-3 in which the frequency of reporting decreased between 2001–2010 (*n* = 34) and 2011–2015 (*n* = 24). Serology was the primary laboratory technique to diagnose DENV throughout the decades (i.e., between 1991 and 2000, it was the only reported technique). Seventy percent of the countries included in this review reported DENV. Interestingly, only Brazil and Cuba reported DENV between 1981 and 1990.

### Arbovirus other than dengue

The most predominant non-DENV arboviruses reported were the Oropouche virus (OROV) (*n* = 16), followed by yellow fever virus (YFV) (*n* = 14), Venezuelan equine encephalitis virus (*n* = 11), and Mayaro virus (*n* = 7). Emergent viral diseases such as Chikungunya (CHIKV) and Zika virus (ZIKV) were also reported. Among adults, the viruses most often reported were YFV and CHIKV. ZIKV was reported in one article in 2015 from Brazil, which demonstrated the first autochthonous transmission of the virus in the southeastern part of the country [8].

The reporting pattern varied over time. CHIKV (*n* = 6) was the most frequently reported arbovirus between 2011 and 2015, while OROV (*n* = 9) predominated during 2001 and 2010, reflecting the occurrence of CHIKV and OROV outbreaks on those years in the Americas [9, 10]. Fig. S9 shows the distribution of the principal non-DENV arboviruses.

### Bacterial infections

The most commonly reported bacterial infections were due to *Staphylococcus* spp. (*n* = 82), *Rickettsia* spp. (*n* = 70), *Leptospira* spp. (*n* = 55), non-*Streptococcus pneumoniae* (*n* = 39), *Bartonella* spp. (*n* = 35), *Streptococcus pneumoniae* (*n* = 28), and *Salmonella* spp. (*n* = 22). Among children, *Streptococcus pneumoniae* and *Salmonella* spp. were common, while in infants, *Staphylococcus* spp. predominated. From 1980 to 1990, the main bacterial infection reported were *Salmonella* spp. (*n* = 6), *Brucella* spp. (*n* = 3), *Bartonella* spp. (*n* = 2), and *Leptospira* spp. (*n* = 2). In contrast, between 2011 and 2015, the main bacterial pathogens reported were *Staphylococcus* spp. (*n* = 48), *Rickettsia* spp. (*n* = 23), and *Bartonella* spp. (*n* = 16).

### Zoonotic bacterial infections

**Fig. 5:**
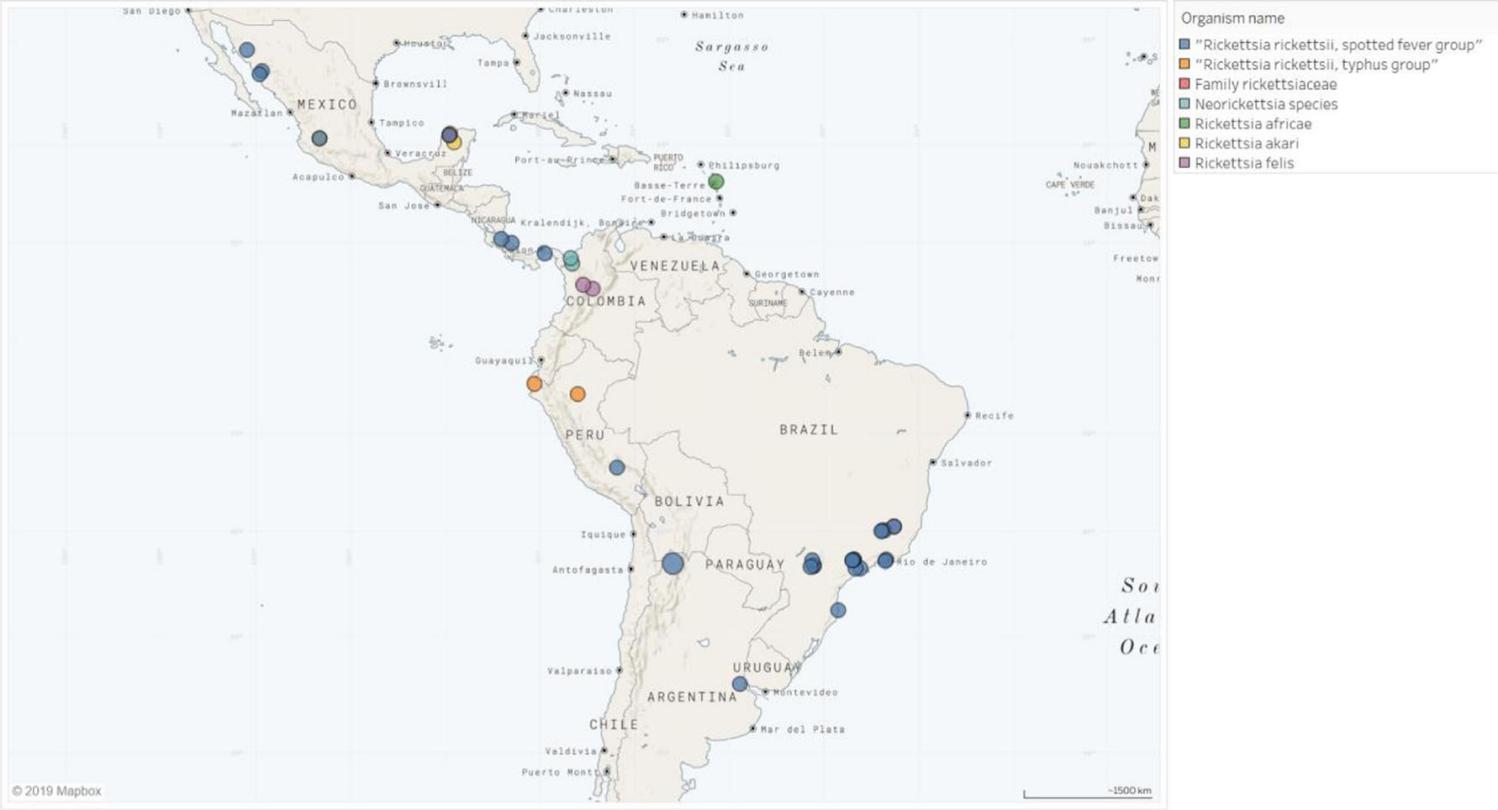
Rickettsiosis in Latin America, 1980–2015. Geographical distribution of study sites where *Rickettsia* spp. has been reported in Latin America

The rickettsia species reported were *Rickettsia rickettsii* (*n* = 35), *Rickettsia typhi* (*n* = 9), *Rickettsia parkeri* (*n* = 5), *Rickettsia felis* (*n* = 4), *Rickettsia akari* (*n* = 3), *Rickettsia prowazekii* (*n* = 1), and *Rickettsia africae* (*n* = 1). Figure 5 shows the distribution of each species throughout the region.

The *Leptospira interrogans* serogroups reported were icterohaemorrhagiae (*n* = 3), pomona (*n* = 2), ballum (*n* = 2), canicola (*n* = 2), bataviae (*n* = 1), cynopteri (*n* = 1), djasiman (*n* = 1), serjroe (*n* = 1), and tarassovi (*n* = 1). Fig. S10 shows the distribution of each serogroup throughout the region.

The *Bartonella* species that were reported causing human diseases in LA were *B*. *bacilliformis* (*n* = 14), *B*. *henselae* (*n* = 13), and *B*. *quintana* (*n* = 1). Fig. S11 shows the distribution of *Bartonella* species in the region.

### Fungal infections

The most predominant reported fungal pathogens were *Histoplasmosis* spp. (*n* = 15), *Candida* spp. (*n* = 7), *Paracoccidioides* spp. (*n* = 7), and *Aspergillus* spp. (*n* = 6). Histoplasmosis was dominant among adults, whereas candidiasis in those of all ages. Paracoccidioidomycosis, also known as Brazilian blastomycosis, were reported in both adults and pediatric patients.

### Parasitic infections

*Leishmania* spp. was the only parasite responsible for febrile illness among infants. In children, the disease was mainly attributed to *Leishmania* spp. (*n* = 4) and *Trypanosoma cruzi* (*n* = 2). Among adults, *Toxoplasma* spp. (*n* = 16), *Leishmania* spp. (*n* = 7), and *Strongyloides* spp. (*n* = 3) were the main etiological agents. Fig. S12 shows the geographical distribution of the main parasitic infection in the region.

## Discussion

The present study summarizes the incomplete data available on pathogens (other than *Plasmodium* spp., HIV, or tuberculosis) associated with febrile illness in studies across LA for over 30 years, during a time where malaria transmission has significantly declined in the region [11]. Furthermore, the work also provides etiology maps, showing evidence of where particular pathogens have been tested for and found to occur.

Our review encompasses etiological fever investigations conducted in every country of LA (predominantly from South America), where we identify a broad range of pathogens that were recognized in usually sterile sources. We found that viral and bacterial diseases accounted for more than 80% of non-malaria febrile illness diagnosis in our setting. Ubiquitous tropical vector-borne viruses (DENV being the most important) and well-known bacterial pathogens (i.e., *Staphylococcus* spp., *Leptospira* spp.) were commonly reported. Interestingly, we identified some underreported pathogens (such as *Salmonella* spp.), with the capacity to induce severe infection in susceptible hosts, that were more frequently reported than generally expected by clinicians in LA [12].

Another important observation was the fact that etiology varied according to patient’s age groups, with viral diseases being regularly detected in patients of all ages and bacterial illness in adults. Studies rarely focus on more than one important pathogen group at a time. Further, the absence of more reports describing ZIKV is explained by the timing restriction criteria imposed. ZIKV created an explosive epidemic in much of the LA starting 2015, with important implications for public health, for people living in endemic regions, and for travelers and their sexual partners [13–16].

### Strengths and weakness

Our review adhered to a rigorous methodological approach considered that three major biomedical databases (with a focus on LA) without language restriction were sought, covering a significant part of the reports published between 1980 and 2015. Besides, this review provides new interactive visualization and maps for public access describing the distribution of pathogens responsible for febrile illness, using a standardized electronically report form.

Our study has limitations. Our etiology maps reflect trends in what was investigated for as much as trends in what was causing febrile illness at the time the data were collected.

Readers might be tempted to estimate incidence and prevalence rates for a particular organism after considering our data. However, due to the underlying heterogeneity between studies, different laboratory techniques, and testing algorithms applied along the three decades, a formal meta-analytic process was deemed inappropriate, limiting comparability between them. The data presented here convey the information that a particular organism has been tested for and found to occur in a specific region. But drawing conclusions about where the same pathogen is not present is misleading. Our study does not distinguish between the absence of a pathogen that was adequately tested for from the absence of data on that particular pathogen because of inadequate detection. For instance, the fact that dengue was the main prevalent pathogen reported associated with febrile illness reflects in part the fact the dengue is hyperendemic in the region and so a common cause for looking for in febrile illness studies. Conversely, other less commonly fungal and parasitic pathogens responsible for febrile illness in LA were substantially underreported because they are not routinely investigated in clinical practice.

Second, the process of attributing causality in each of the febrile episodes included in this review is challenging, considering the inherent limitation of the laboratory methods described. For instance, viral illness was diagnosed by either serology or molecular methods. The results of serology are problematic mainly if only acute sera were performed, as immunoglobulin persists for some time after an acute episode. On the other hand, the diagnostic performance of molecular methods varies according to pathogen group and time of presentation [17]. Laboratory data should, therefore, always be interpreted in the context of the clinical presentation of patients, and fever etiology studies should be carefully designed to avoid overestimation of the prevalence and the public health importance of specific pathogens.

### Implications for policymakers and other stakeholders

We argue that more focus needs to be given to the extensive improvements of epidemiologic surveillance systems at the national and international levels. Additional support should be provided for the implementation of new diagnostics, antimicrobials, and vaccines and improved stewardship of existing antimicrobials to avoid further selection and emergence of resistant bacteria. Data sources from public and private sectors should be integrated and contain sufficient information on individuals’ patients and their outcomes to use this information to support surveillance and data collection efforts, as well as to inform fever management algorithms.

As pointed out in our results, DENV was the main pathogen reported and associated with NMFI in LA, and as such, future studies should provide a better characterization of the epidemiological distribution of this arbovirus in our region.

Protocols and data collection mechanisms need to be standardized to allow an accurate depiction of the real health burden of NMFI. The problems are exacerbated in low- and middle-income countries, where there is often inadequate surveillance and minimal laboratory capacity. The lack of consistency in the measurement and reporting of fever etiology data also makes it difficult to compare findings among different countries, and sometimes even within one country.

Our etiology maps might have implications for clinicians providing care to international travelers interested in visiting LA. Travelers should be informed about the risks of the infectious diseases associated at a country-specific level, including recommendations on vaccine requirements and antimicrobial prophylaxis.

Finally, the maps identify research gaps within particular areas in the region where more resources should be allocated in order to inform public health policy for fever epidemiology and management. For instance, much of the Midwestern part of Brazil and the South and Southeast of Argentina were underrepresented, limiting the generalization of our findings to these areas. These maps would be useful to prioritize locations with the most significant gaps where focus capacity development initiatives should be in place.

## Conclusion

In summary, this mapping exercise of febrile illness etiology in LA demonstrated substantial heterogeneity, inconsistent reporting, and areas with considerable gaps where more resources should be allocated and capacity development initiatives put in place. The etiology of fever in LA was predominantly attributed to viruses or bacteria, being DENV the most frequently reported pathogen. Our findings (i) highlight the need to standardize protocols and reporting guidelines of fever etiology studies for better comparability of results and improved interpretation, (ii) improve existing epidemiological surveillance networks with data collection mechanisms to inform global fever policy priorities, and (iii) prioritize the development of pathogen-targeted diagnostics and implementation of tailored syndromic fever-testing algorithms in the region.

## Supplementary information

**Additional file 1: Fig. S1.** PRISMA Flow Diagram. Flow diagram of reviewed studies. Numbers of studies screened, assessed for eligibility, and included in the review, with reasons for exclusions. **Fig. S2.** Number of publications by year. Legend: Schematic representation of the number of included articles per year. **Fig. S3.** Location of study sites and number of studies per site. Geographic location and number of studies per site included in the review. **Fig. S4.** Study type per country. Study types according to country of publication. **Fig. S5.** Sample sources over time. Legend: Type of specimen reported in the included studies throughout time. **Fig. S6.** Distribution of the main pathogen groups. Geographical distribution of the main pathogen groups reported in Latin America. **Fig. S7.** Dengue distribution according to age category. Dengue distribution according to age groups. **Fig. S8.** Dengue serotypes. Geographical distribution of dengue serotypes in Latin America. Note that in some reports, dengue serotype was not mentioned and, in those cases, we referred as “DENV”. **Fig. S9.** Distribution of non-dengue arboviruses in Latin America. Geographical distribution of the main arboviruses other than dengue in Latin America. **Fig. S10.** Leptospirosis in Latin America. Geographical distribution of the main Leptospira spp. reported in the included studies in Latin America. **Fig. S11.** Bartonellosis in Latina America. Geographical distribution of the main *Bartonella* spp. reported in the included studies in Latin America. **Fig. S12.** Distribution of the main parasitic infections in Latin America. Geographical distribution of the main parasitic infections reported in the included studies in Latin America.

**Additional file 2.**

## Data Availability

The datasets analyzed during the current study are available in the Non-malaria febrile illness map’s website, collated by Infectious Diseases Data Observatory (IDDO) and hosted in WWARN server.

## Abbreviations

CHIKV: Chikungunya virus
DENV: Dengue virus
DENV-1: Dengue serotype 1
DENV-2: Dengue serotype 2
DENV-3: Dengue serotype 3
DENV-4: Dengue serotype 4
LA: Latin America
NMFI: Non-malaria febrile illness
OROV: Oropouche virus
YFV: Yellow fever virus
ZIKV: Zika virus
