# Supplementary material for: When fever is not malaria in Latin America: a systematic review
Source: BMC Med. 2020 Sep 21;18:294. doi: 10.1186/s12916-020-01746-z (PMC7504635; doi:10.1186/s12916-020-01746-z)
Supplement: Supplementary file 1 — Additional file 1: Fig. S1. PRISMA Flow Diagram. Flow diagram of reviewed studies. Numbers of studies screened, assessed for eligibility, and included in the review, with reasons for exclusions. Fig. S2. Number of publications by year. Legend: Schematic representation of the number of included articles per year. Fig. S3. Location of study sites and number of studies per site. Geographic location and number of studies per site included in the review. Fig. S4. Study type per country. Study types according to country of publication. Fig. S5. Sample sources over time. Legend: Type of specimen reported in the included studies throughout time. Fig. S6. Distribution of the main pathogen groups. Geographical distribution of the main pathogen groups reported in Latin America. Fig. S7. Dengue distribution according to age category. Dengue distribution according to age groups. Fig. S8. Dengue serotypes. Geographical distribution of dengue serotypes in Latin America. Note that in some reports, dengue serotype was not mentioned and, in those cases, we referred as “DENV”. Fig. S9. Distribution of non-dengue arboviruses in Latin America. Geographical distribution of the main arboviruses other than dengue in Latin America. Fig. S10. Leptospirosis in Latin America. Geographical distribution of the main Leptospira spp. reported in the included studies in Latin America. Fig. S11. Bartonellosis in Latina America. Geographical distribution of the main Bartonella spp. reported in the included studies in Latin America. Fig. S12. Distribution of the main parasitic infections in Latin America. Geographical distribution of the main parasitic infections reported in the included studies in Latin America. [file 12916_2020_1746_MOESM1_ESM.docx]

Supplementary Figure 1. PRISMA Flow Diagram


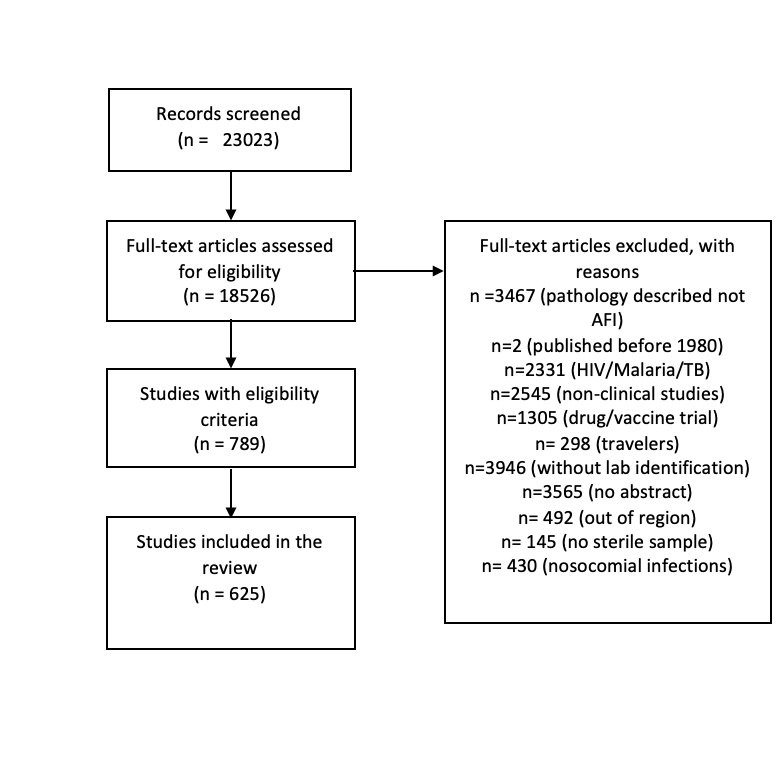


Legend: Flow diagram of reviewed studies. Numbers of studies screened, assessed for eligibility, and included in the review, with reasons for exclusions.

Supplementary Figure 2. Number of publications by year


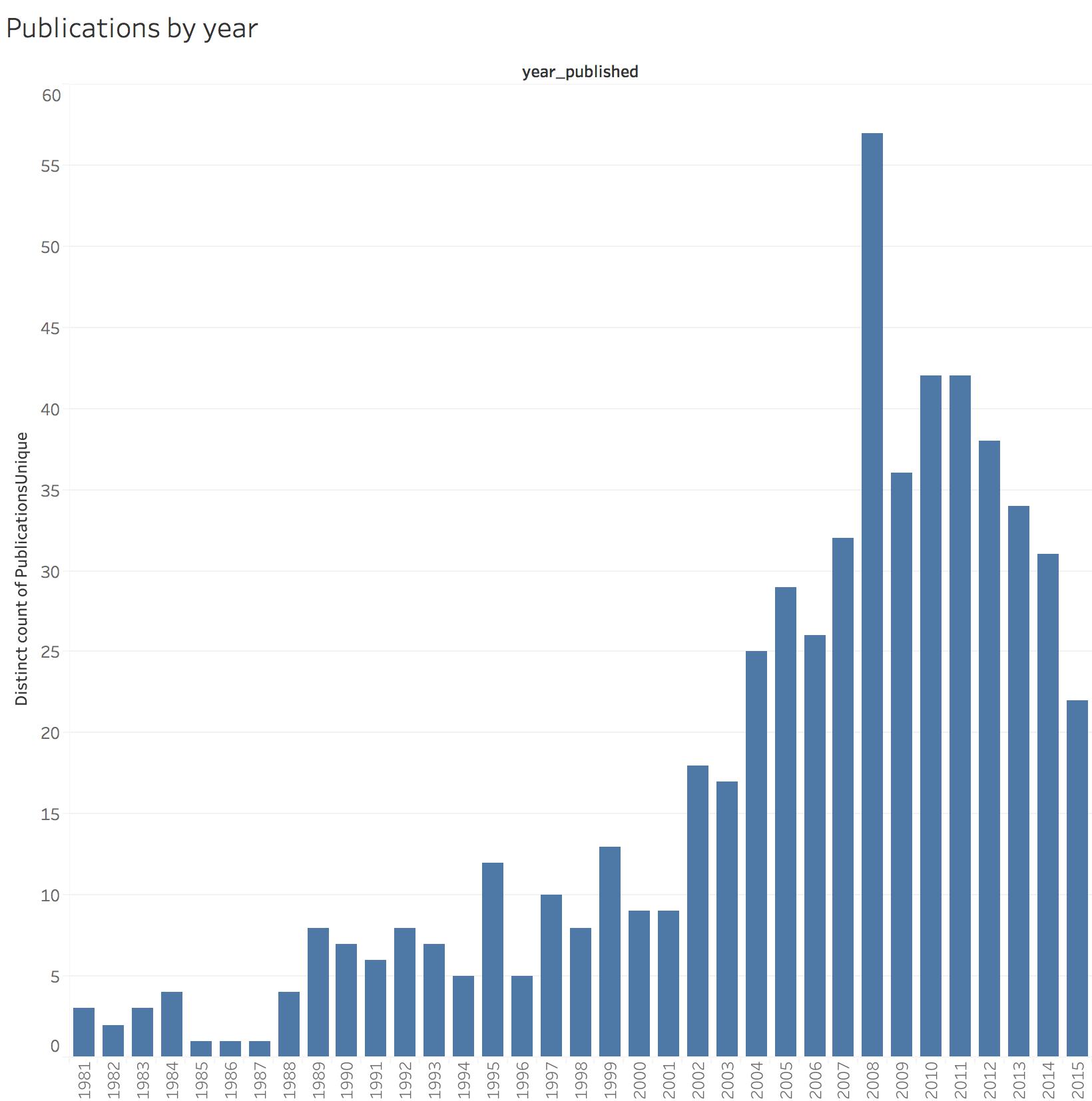


Legend: Schematic representation of the number of included articles per year.

Supplementary Figure 3. Location of study sites and number of studies per site


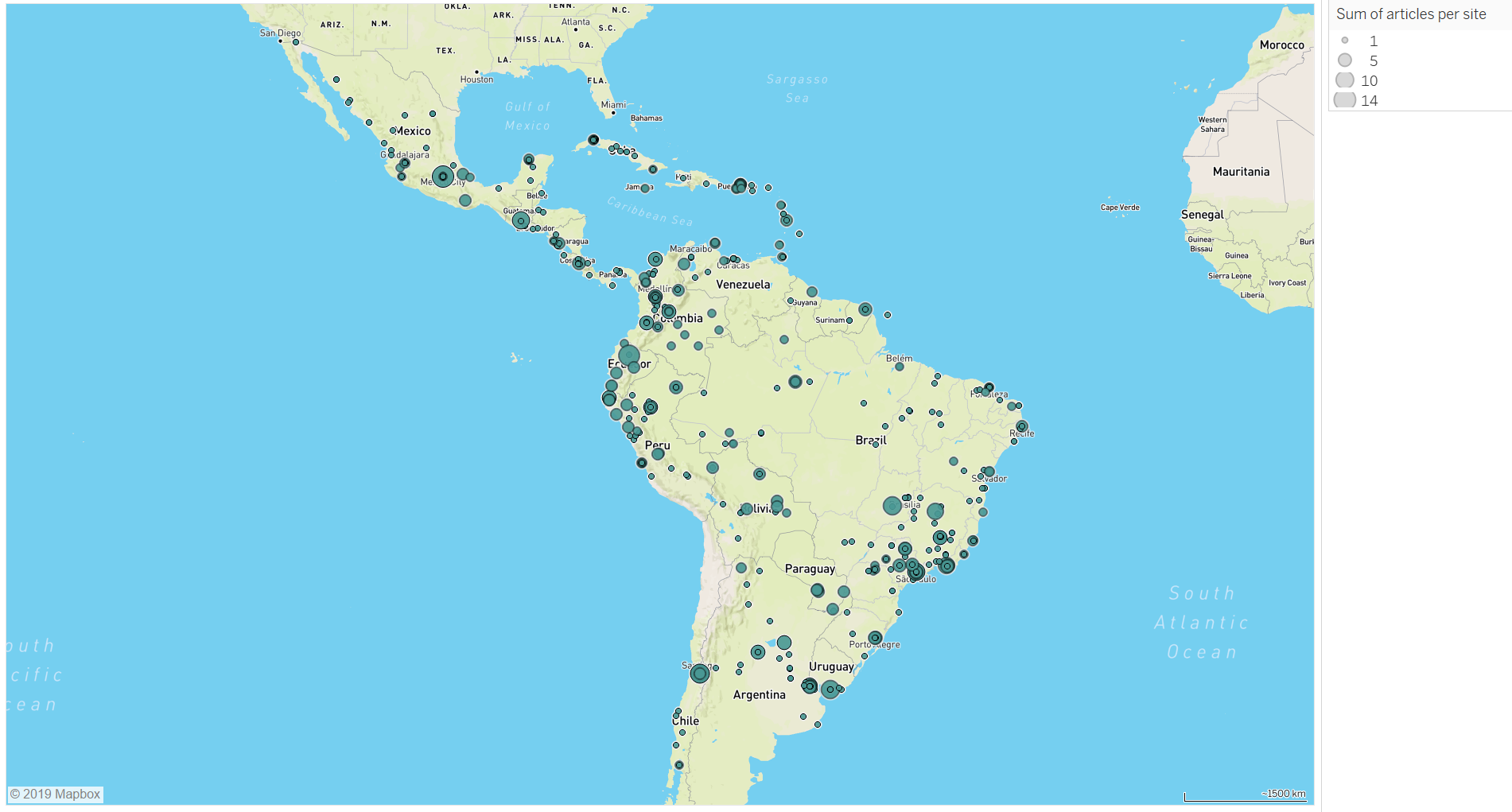


Legend: Geographic location and number of studies per site included in the review.

Supplementary Figure 4. Study type per country


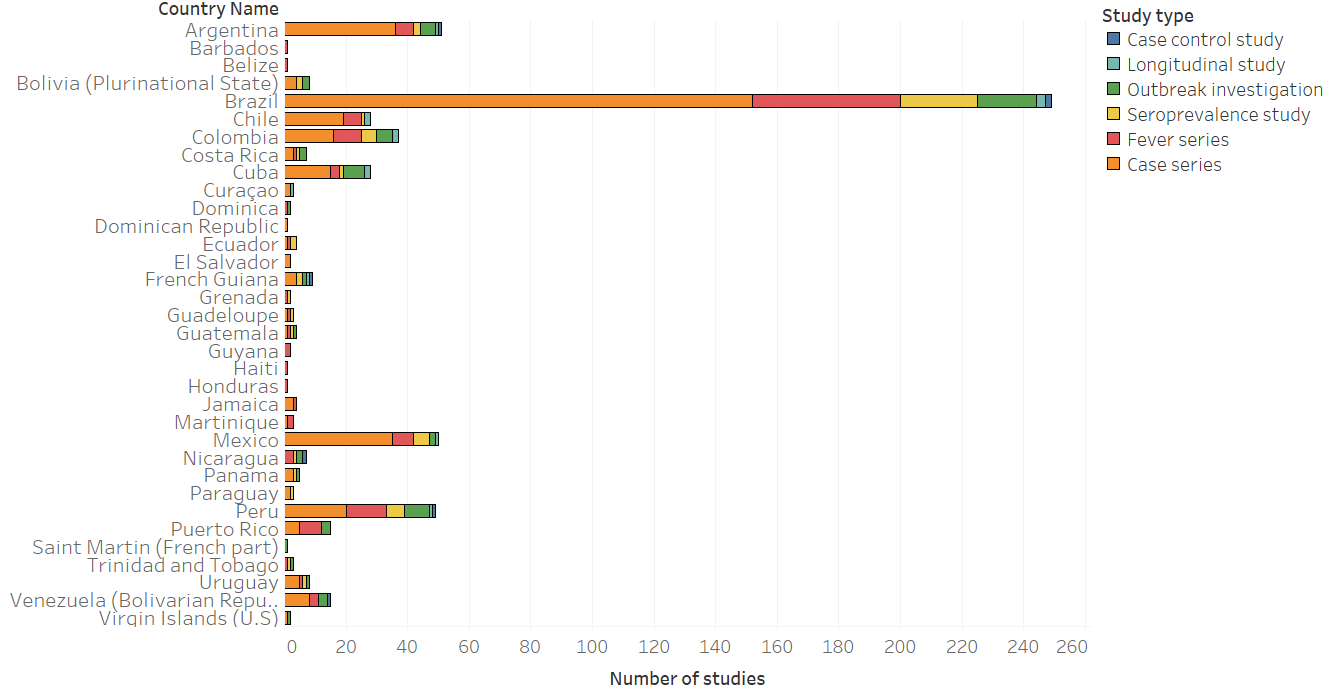


Legend: Study types according to country of publication.

Supplementary Figure 5. Sample sources over time


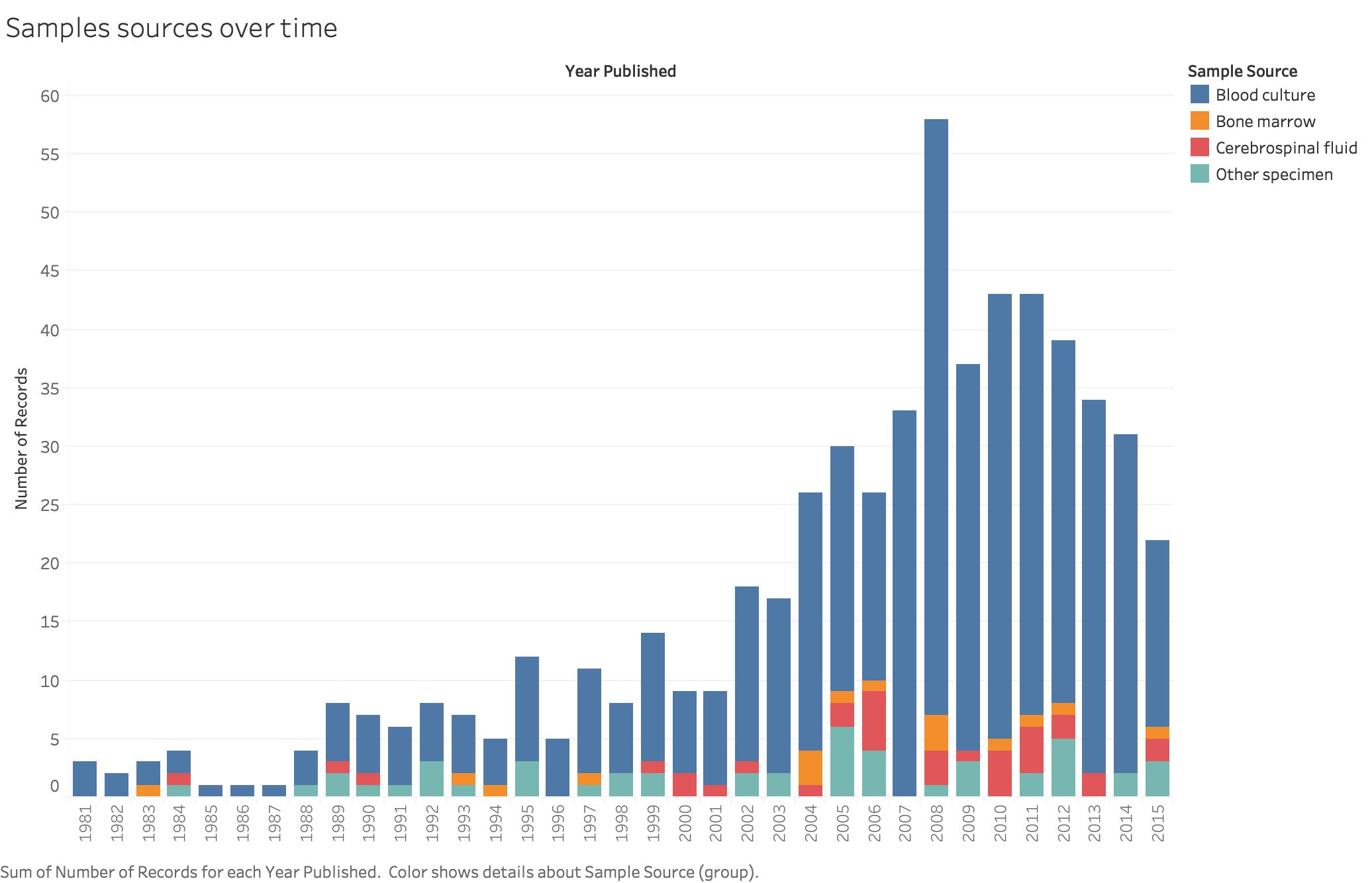


Legend: Type of specimen reported in the included studies throughout time.

Supplementary Figure 6. Distribution of the main pathogen groups


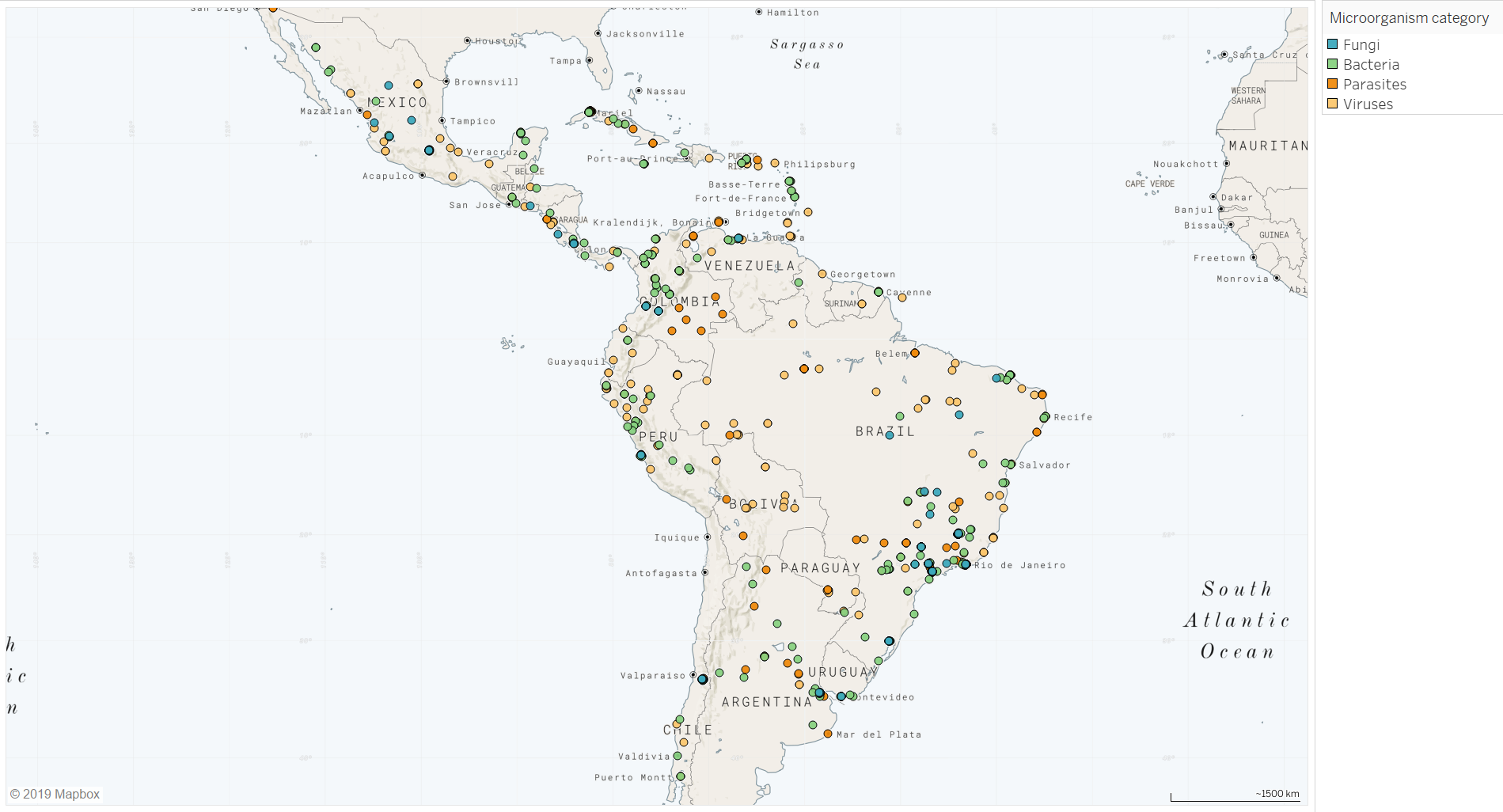


Legend: Geographical distribution of the main pathogen groups reported in Latin America.

Supplementary Figure 7. Dengue distribution according to age category


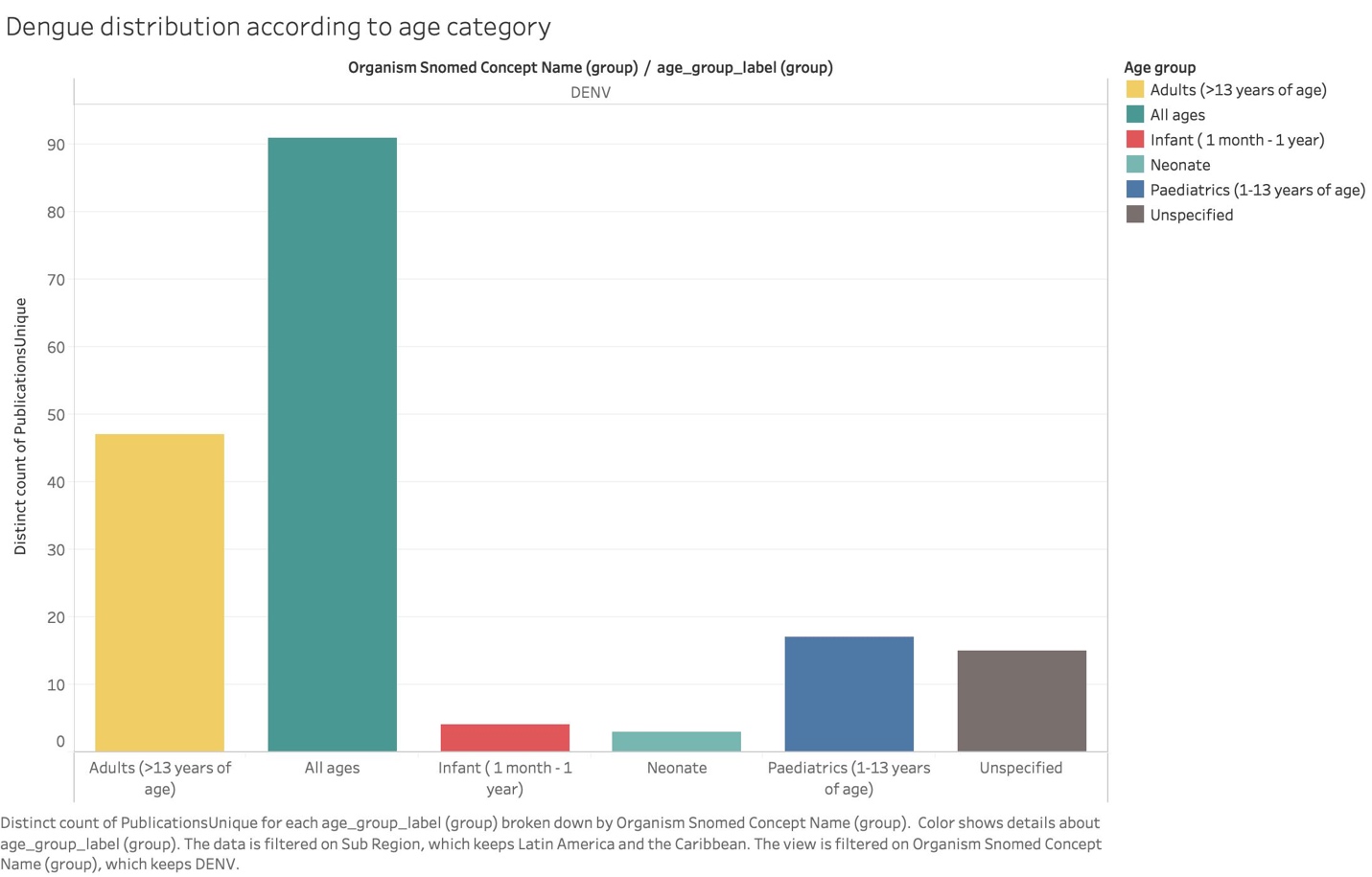


Legend: Dengue distribution according to age groups.

Supplementary Figure 8. Dengue serotypes


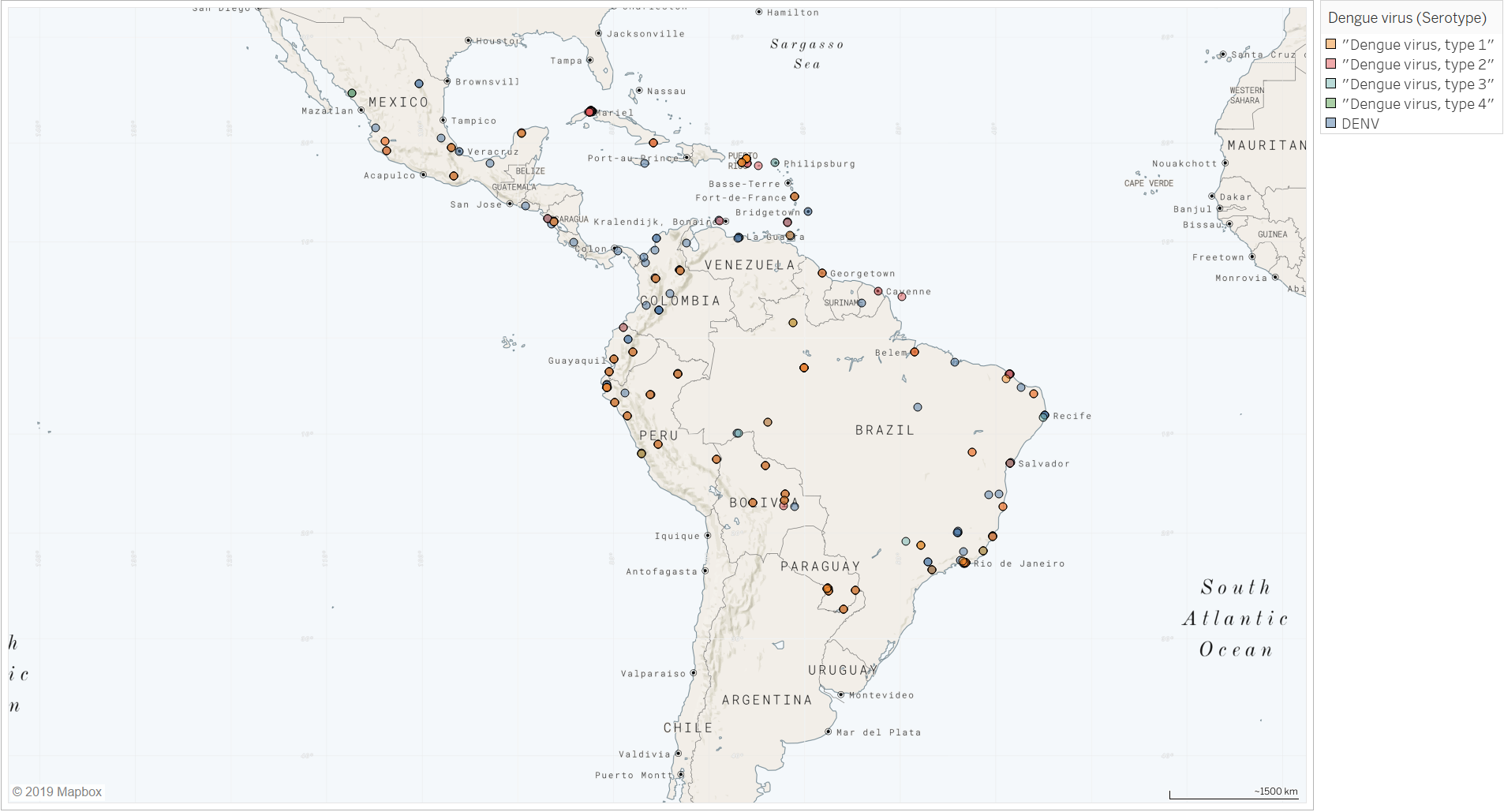


Legend: Geographical distribution of dengue serotypes in Latin America. Note that in some reports, dengue serotype was not mentioned and, in those cases, we referred as “DENV”.

Supplementary Figure 9. Distribution of non-dengue arboviruses in Latin America


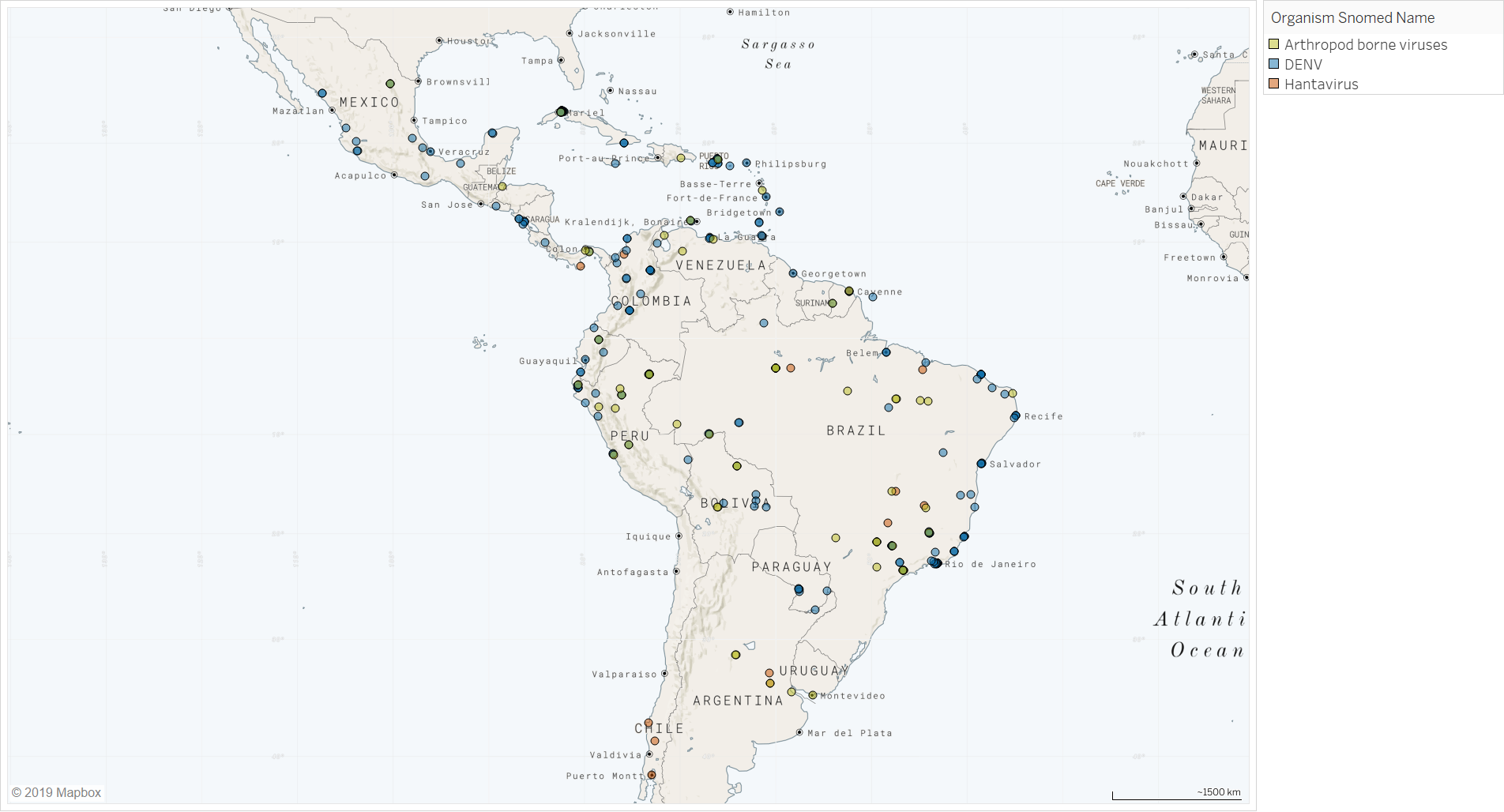


Legend: Geographical distribution of the main arboviruses other than dengue in Latin America.

Supplementary Figure 10. Leptospirosis in Latin America


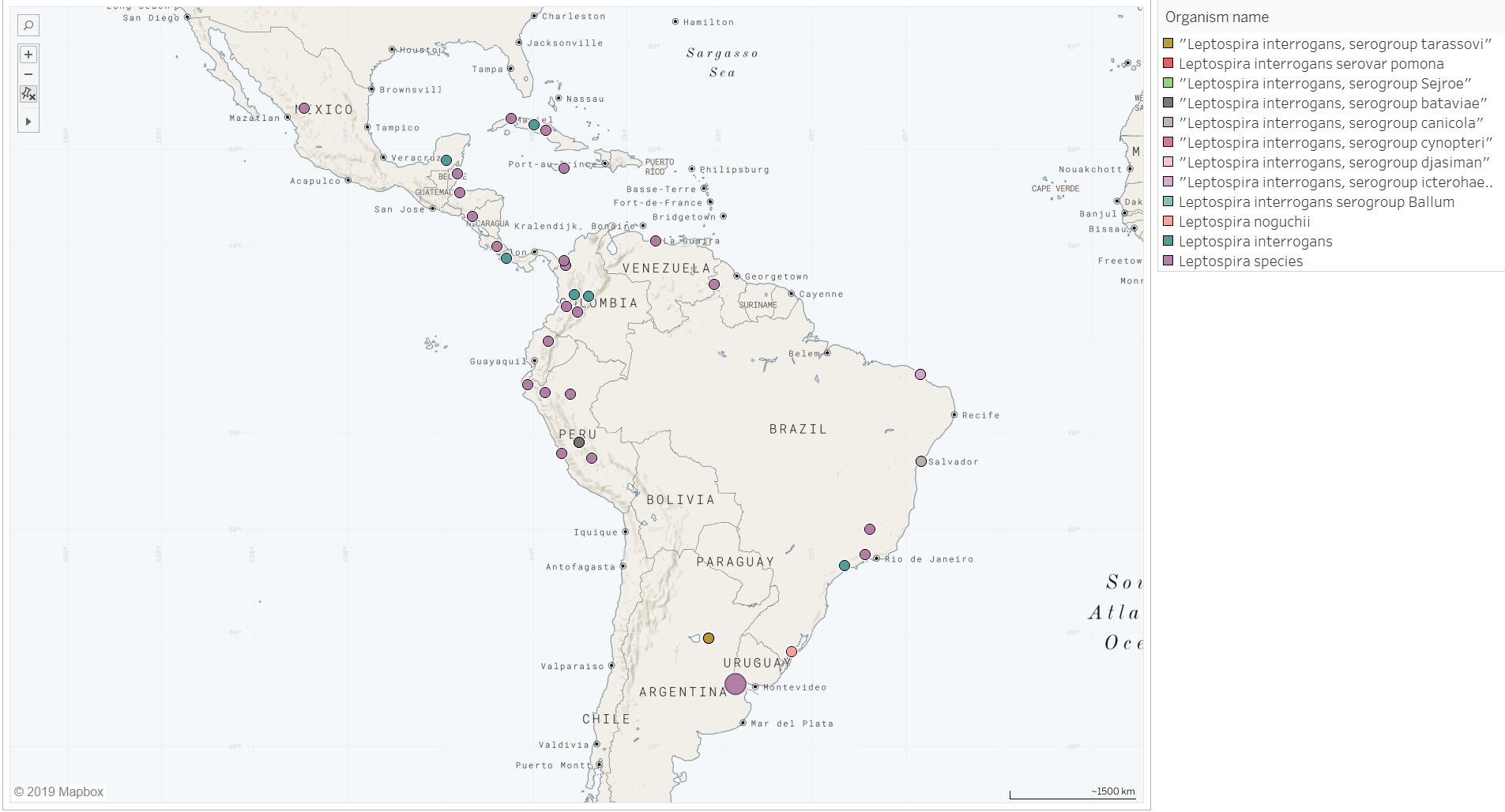


Legend: Geograhical distrubution of the main Leptospira spp reported in the included studies in Latin America.

Supplementary Figure 11. Bartonellosis in Latina America


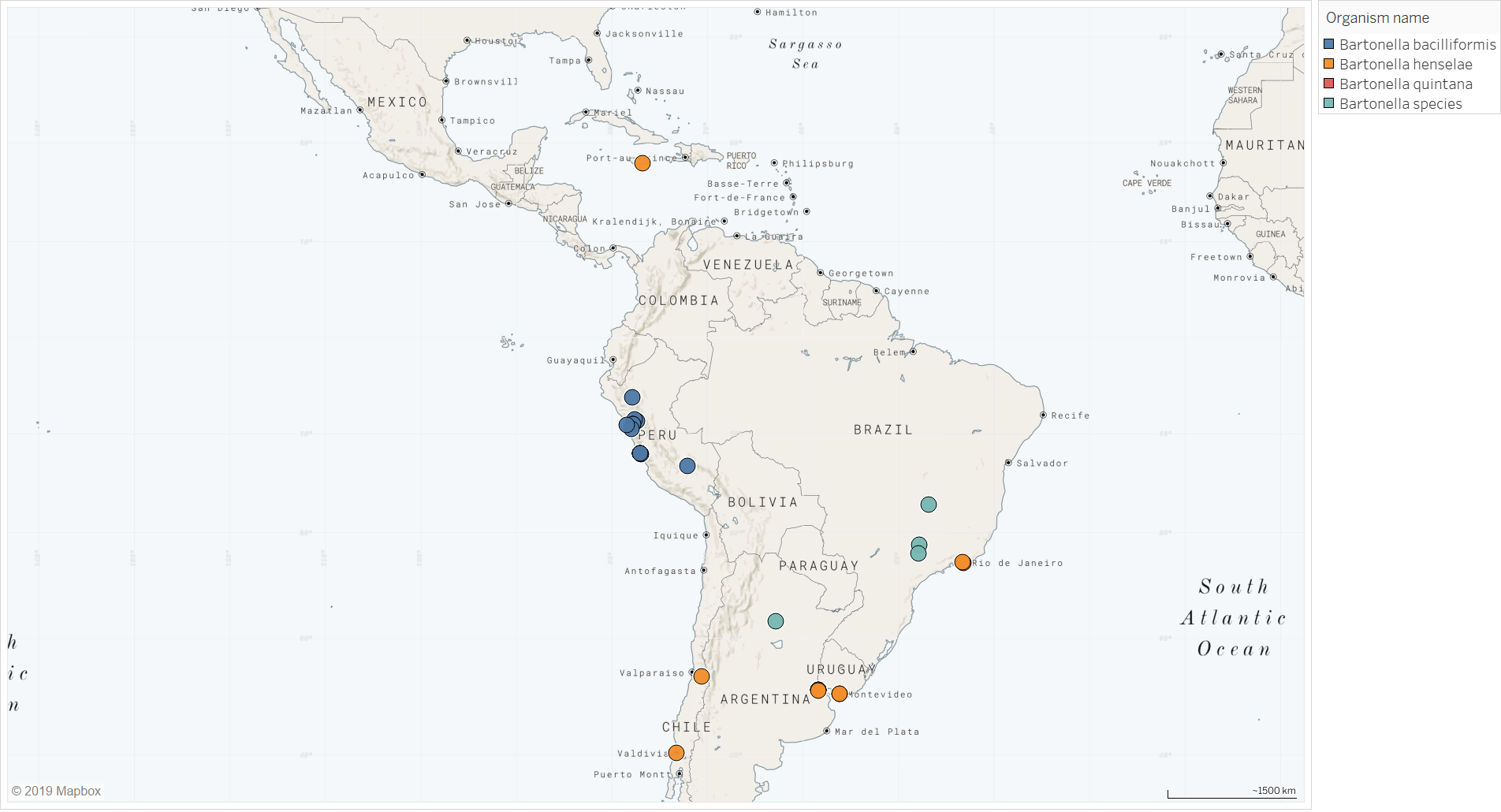


Legend: Geograhical distrubution of the main Bartonella spp reported in the included studies in Latin America.

Supplementary Figure 12. Distribution of the main parasitic infections in Latin America


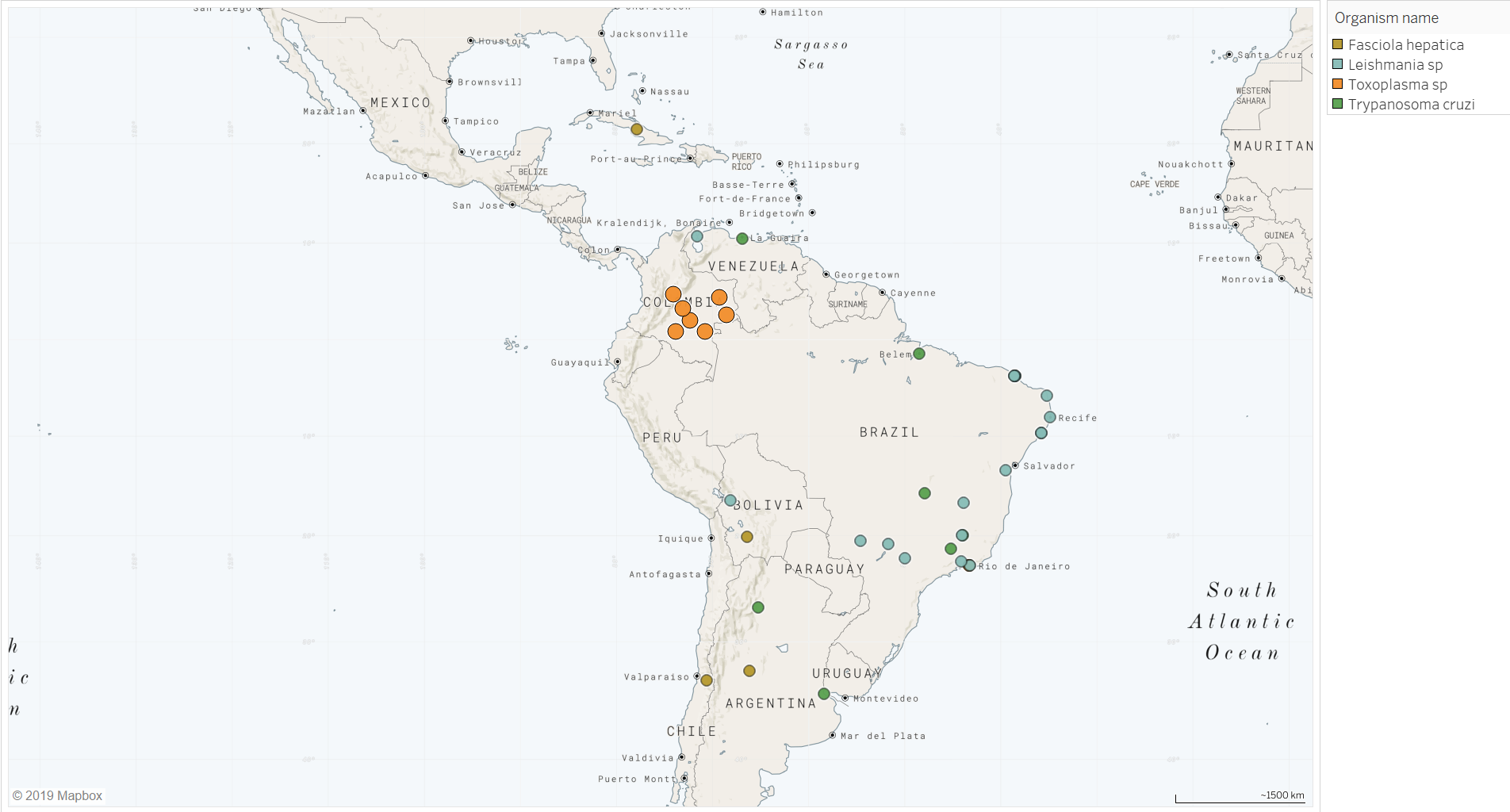


Legend: Geograhical distrubution of the main parasitic infections reported in the included studies in Latin America.
