# Supplementary material for: When fever is not malaria in Latin America: a systematic review
Source: BMC Med. 2020 Sep 21;18:294. doi: 10.1186/s12916-020-01746-z (PMC7504635; doi:10.1186/s12916-020-01746-z)
Supplement: Supplementary file 2 — Additional file 2. [file 12916_2020_1746_MOESM2_ESM.doc]

1. **Americas - Ovid MEDLINE(R) In-Process & Other Non-Indexed Citations, Ovid MEDLINE(R) Daily, Ovid MEDLINE(R) and Ovid OLDMEDLINE(R) <1946 to Present> - Searched 13^th^ July 2015**

1 exp Anti-Bacterial Agents/dt, pd, tu, th, ut [Drug Therapy, Pharmacology, Therapeutic Use, Therapy, Utilization] (396988)

2 exp Bacteremia/ or (bacteremi* or bacteraemi* or septicemi* or septicaemi*).ti,ab. (42134)

3 exp Sepsis/ or ("blood stream infection*" or "bloodstream infection*" or "blood stream pathogen*" or "bloodstream pathogen*" or (("blood borne" or bloodborne or blood-borne) adj (infection* or pathogen*))).ti,ab. (103158)

4 exp Fever/ or (febrile or fever* or pyrexia or pyrexic or pyrexial or pyrexiae or hyperthermi* or (raised adj2 temperature) or (high adj 2 temperature) or (elevated adj2 temperature)).ti,ab. (194090)

5 or/1-3 (501691)

6 (babesi* or leishmania* or chagas or trypanosom* or "sleeping sickness").ti,ab. (61688)

7 Babesia microti/ (225)

8 exp Trypanosoma/ (21833)

9 exp Trypanosomiasis/ not Trypanosomiasis, Bovine/ (17824)

10 exp Leishmania/ (16157)

11 exp Leishmaniasis/ (18280)

12 Influenza A Virus, H1N1 Subtype/ or Influenza A virus/ or Influenza a virus.ti,ab. (31204)

13 exp Arbovirus infections/ or Arboviruses/ or exp Encephalitis viruses, Japanese/ or Encephalitis virus, Japanese/ or Encephalitis virus, Murray Valley/ or "Encephalitis virus, St. Louis"/ or West Nile virus/ or Encephalitis viruses, tick-borne/ or Yellow fever virus/ or (Japanese Encephalitis or Japanese B Encephalitis or Phlebotomus fever or Rift Valley fever or Yellow fever or ((California or "St Louis" or Tick-borne) adj Encephalitis) or Kyasanur Forest disease).ti,ab. (37910)

14 Chikungunya virus/ or Chikungunya.ti,ab. (2362)

15 exp Epstein-Barr Virus Infections/ or Epstein-Barr.ti,ab. (41647)

16 exp Dengue/ or Dengue Virus/ or Dengue.ti,ab. (14155)

17 Respiratory Syncytial Virus, Human/ or Respiratory Syncytial Virus Infections/ or (Respiratory Syncytial Virus or RSV).ti,ab. (13604)

18 Measles/ or Measles virus/ or Measles.ti,ab. (23006)

19 Hantavirus/ or Hantaan Virus/ or Puumala Virus/ or Seoul Virus/ or Sin Nombre Virus/ or Hantavirus Infections/ or Hantavirus Pulmonary Syndrome/ or Hemorrhagic Fever with Renal Syndrome/ or (Hantavirus or Hantaan or Puumula or Seoul virus or Sin Nombre virus or Hemorrhagic fever or Haemorrhagic fever).ti,ab. (10240)

20 Nipah Virus/ or Nipah Virus.ti,ab. (592)

21 Coxsackievirus Infections/ or (Coxsackie or Coxsackievirus*).ti,ab. (8652)

22 Cytomegalovirus Infections/ or Cytomegalovirus/ or Cytomegalovirus*.ti,ab. (42714)

23 Hepatitis/ or Hepatitis, Viral, human/ or Hepatitis a/ or Hepatitis b/ or Hepatitis b, chronic/ or Hepatitis c/ or Hepatitis c, chronic/ or Hepatitis e/ or Hepatitis b virus/ or Hepacivirus/ or Hepatitis e virus/ or Hepatitis a virus/ or Hepatitis a virus, human/ or (Hepatitis adj (a or b or c or e)).ti,ab. (174316)

24 (Coxiella burnetii or Salmonella typhi or Salmonella paratyphi or Burkholderia pseudomallei or Pseudomonas pseudomallei or Brucella or Escherichia coli or E-coli or E coli or Citrobacter freundii or Listeria monocytogenes or (Leptospira adj (kmetyi or interogans or weilii or parva)) or Rickettsia or Orientia tsutsugamushi or mycobacterium tuberculosis or h1n1 or Human Herpesvirus 4 or Human Herpes virus 4 or Ehrlichia or Anaplasma or Bartonella or Borrelia or Neorickettsia or Sennetsu or Blastomyces or Cryptococcus or Coccidioides or Histoplasma or Penicillium marneffii or Talaromyces or Yersinia pestis or Francisella tularensis or Klebsiella pneumoniae or Mycoplasma).ti,ab. (392875)

25 (Q fever or salmonellosis or salmonella infection* or melioidosis or brucellosis or listeria infection* or listeriosis or leptospira* infection* or leptospirosis or rickettsia* infection* or rickettsios* or enteric fever or typhoid fever or paratyphoid fever or plague or anaplasmosis or ehrlichiosis or cat scratch fever or cat scratch disease or trench fever or Carrion* disease or borreliosis or lyme or bartonellosis or relapsing fever or typhus or blastomycosis or cryptococcal or coccidioidomycosis or valley fever or histoplasmosis or penicilliosis or tularaemia or tularemia).ti,ab. (79926)

26 Blood-Borne Pathogens/ (2796)

27 ((gram-positive adj3 (bacteria* or infection*)) or (gram-negative adj3 (bacteria* or infection*))).ti,ab. (41914)

28 Cerebrospinal Fluid/ or (cerebrospinal fluid or csf).ti,ab. (125993)

29 exp Bacterial Infections/ not exp Bacteremia/ (745719)

30 exp Bacteria/ (1126988)

31 or/6-30 (2184417)

32 exp Animals/ (18313008)

33 Humans/ (14228720)

34 32 not (32 and 33) (4084288)

35 (Bolivia or El Salvador or Guatemala or Guyana or Honduras or Nicaragua or Paraguay or Argentina or Belize or Brazil or Colombia or Costa Rica or Cuba or Dominica or Dominican Republic or Ecuador or French Guiana or Grenada or Guadeloupe or Haiti or Jamaica or Martinique or Mexico or Montserrat or Panama or Peru or St Lucia or Saint Lucia or Grenadines or Suriname or Surinam or Venezuela or Anguilla or Antigua or Barbuda or Aruba or Bahamas or Barbados or Bermuda or Cayman Islands or Chile or Curacao or Antilles or Puerto Rico or St Martin or Sint Maarten or (Trinidad adj2 Tobago) or (Turks adj2 Caicos Islands) or Virgin Islands or Uruguay or ((St Kitts or Saint Kitts) adj2 Nevis)).ti,ab,cp. (336351)

36 west indies/ or "antigua and barbuda"/ or bahamas/ or barbados/ or british virgin islands/ or cuba/ or dominica/ or dominican republic/ or grenada/ or guadeloupe/ or haiti/ or jamaica/ or martinique/ or netherlands antilles/ or puerto rico/ or "saint kitts and nevis"/ or saint lucia/ or "saint vincent and the grenadines"/ or "trinidad and tobago"/ or united states virgin islands/ or belize/ or costa rica/ or el salvador/ or guatemala/ or honduras/ or nicaragua/ or panama/ or panama canal zone/ or argentina/ or bolivia/ or brazil/ or chile/ or colombia/ or ecuador/ or guyana/ or paraguay/ or peru/ or uruguay/ or venezuela/ (131335)

37 caribbean region/ or central america/ or latin america/ or south america/ (18506)

38 (caribbean or west indies or central america or latin america or south america).ti,ab. (29486)

39 35 or 36 or 37 or 38 (391511)

40 4 and 39 (6787)

41 5 and 31 and 39 (6923)

42 40 or 41 (13222)

43 42 not 34 (11993)

44 limit 43 to (yr="1980 -Current" and (chinese or english or french or portuguese or spanish)) (10508)

1. **VHL (Bireme) – Latin-American health databases – LILACS, IBECS, PAHO, CUMED, MedCarib - Searched 10th July 2015**

(ti:(febrile or fever$ or pyrexia or pyrexic or pyrexial or pyrexiae or hyperthermia$ or (raised AND temperature) or (high AND temperature) or (elevated AND temperature) or febril OR febre$ OR pirexia OR pirético OR pirética OR febril OR pyrexiae OR hipertermia$ OR (temperatura AND aumentada) OR (temperatura AND alta) OR (temperatura AND elevada) or febril OR fiebre$ OR pirexia OR pirético OR pirética OR febril OR pyrexiae OR hipertermia$ OR (temperatura AND aumentada) OR (temperatura AND alta) OR (temperatura AND elevada))) OR (mh:(Fever OR "Fever of Unknown Origin")) – 4730 hits combined (4522 duplicates removed in Endnote)

1. **Americas/Caribbean: Embase <1980 to 2015 Week 31> - Searched 4th August 2015**

1 exp Anti-Bacterial Agents/dt, pd, tu, th, ut [Drug Therapy, Pharmacology, Therapeutic Use, Therapy, Utilization] (977338)

2 exp Bacteremia/ or (bacteremi* or bacteraemi* or septicemi* or septicaemi*).ti,ab. (47500)

3 exp Sepsis/ or ("blood stream infection*" or "bloodstream infection*" or "blood stream pathogen*" or "bloodstream pathogen*" or (("blood borne" or bloodborne or blood-borne) adj (infection* or pathogen*))).ti,ab. (188833)

4 exp Fever/ or (febrile or fever* or pyrexia or pyrexic or pyrexial or pyrexiae or hyperthermi* or (raised adj2 temperature) or (high adj 2 temperature) or (elevated adj2 temperature)).ti,ab. (301726)

5 or/1-3 (1132228)

6 (babesi* or leishmania* or chagas or trypanosom* or "sleeping sickness").ti,ab. (64426)

7 Babesia microti/ (669)

8 exp Trypanosoma/ (26002)

9 exp Trypanosomiasis/ not Trypanosomiasis, Bovine/ (20548)

10 exp Leishmania/ (21118)

11 exp Leishmaniasis/ (22954)

12 Influenza A Virus, H1N1 Subtype/ or Influenza A virus/ or Influenza a virus.ti,ab. (29005)

13 exp Arbovirus infections/ or Arboviruses/ or exp Encephalitis viruses, Japanese/ or Encephalitis virus, Japanese/ or Encephalitis virus, Murray Valley/ or "Encephalitis virus, St. Louis"/ or West Nile virus/ or Encephalitis viruses, tick-borne/ or Yellow fever virus/ or (Japanese Encephalitis or Japanese B Encephalitis or Phlebotomus fever or Rift Valley fever or Yellow fever or ((California or "St Louis" or Tick-borne) adj Encephalitis) or Kyasanur Forest disease).ti,ab. (904957)

14 Chikungunya virus/ or Chikungunya.ti,ab. (2955)

15 exp Epstein-Barr Virus Infections/ or Epstein-Barr.ti,ab. (40990)

16 exp Dengue/ or Dengue Virus/ or Dengue.ti,ab. (18610)

17 Respiratory Syncytial Virus, Human/ or Respiratory Syncytial Virus Infections/ or (Respiratory Syncytial Virus or RSV).ti,ab. (15666)

18 Measles/ or Measles virus/ or Measles.ti,ab. (26329)

19 Hantavirus/ or Hantaan Virus/ or Puumala Virus/ or Seoul Virus/ or Sin Nombre Virus/ or Hantavirus Infections/ or Hantavirus Pulmonary Syndrome/ or Hemorrhagic Fever with Renal Syndrome/ or (Hantavirus or Hantaan or Puumula or Seoul virus or Sin Nombre virus or Hemorrhagic fever or Haemorrhagic fever).ti,ab. (11474)

20 Nipah Virus/ or Nipah Virus.ti,ab. (881)

21 Coxsackievirus Infections/ or (Coxsackie or Coxsackievirus*).ti,ab. (8905)

22 Cytomegalovirus Infections/ or Cytomegalovirus/ or Cytomegalovirus*.ti,ab. (56377)

23 Hepatitis/ or Hepatitis, Viral, human/ or Hepatitis a/ or Hepatitis b/ or Hepatitis b, chronic/ or Hepatitis c/ or Hepatitis c, chronic/ or Hepatitis e/ or Hepatitis b virus/ or Hepacivirus/ or Hepatitis e virus/ or Hepatitis a virus/ or Hepatitis a virus, human/ or (Hepatitis adj (a or b or c or e)).ti,ab. (247458)

24 (Coxiella burnetii or Salmonella typhi or Salmonella paratyphi or Burkholderia pseudomallei or Pseudomonas pseudomallei or Brucella or Escherichia coli or E-coli or E coli or Citrobacter freundii or Listeria monocytogenes or (Leptospira adj (kmetyi or interogans or weilii or parva)) or Rickettsia or Orientia tsutsugamushi or mycobacterium tuberculosis or h1n1 or Human Herpesvirus 4 or Human Herpes virus 4 or Ehrlichia or Anaplasma or Bartonella or Borrelia or Neorickettsia or Sennetsu or Blastomyces or Cryptococcus or Coccidioides or Histoplasma or Penicillium marneffii or Talaromyces or Yersinia pestis or Francisella tularensis or Klebsiella pneumoniae or Mycoplasma).ti,ab. (409004)

25 (Q fever or salmonellosis or salmonella infection* or melioidosis or brucellosis or listeria infection* or listeriosis or leptospira* infection* or leptospirosis or rickettsia* infection* or rickettsios* or enteric fever or typhoid fever or paratyphoid fever or plague or anaplasmosis or ehrlichiosis or cat scratch fever or cat scratch disease or trench fever or Carrion* disease or borreliosis or lyme or bartonellosis or relapsing fever or typhus or blastomycosis or cryptococcal or coccidioidomycosis or valley fever or histoplasmosis or penicilliosis or tularaemia or tularemia).ti,ab. (81059)

26 Blood-Borne Pathogens/ (1684)

27 ((gram-positive adj3 (bacteria* or infection*)) or (gram-negative adj3 (bacteria* or infection*))).ti,ab. (48222)

28 Cerebrospinal Fluid/ or (cerebrospinal fluid or csf).ti,ab. (168254)

29 exp Bacterial Infections/ not exp Bacteremia/ (726562)

30 exp Bacteria/ (1221663)

31 or/6-30 (2882681)

32 exp Animals/ (20161866)

33 Humans/ (16020889)

34 32 not (32 and 33) (4140977)

35 (Bolivia or El Salvador or Guatemala or Guyana or Honduras or Nicaragua or Paraguay or Argentina or Belize or Brazil or Colombia or Costa Rica or Cuba or Dominica or Dominican Republic or Ecuador or French Guiana or Grenada or Guadeloupe or Haiti or Jamaica or Martinique or Mexico or Montserrat or Panama or Peru or St Lucia or Saint Lucia or Grenadines or Suriname or Surinam or Venezuela or Anguilla or Antigua or Barbuda or Aruba or Bahamas or Barbados or Bermuda or Cayman Islands or Chile or Curacao or Antilles or Puerto Rico or St Martin or Sint Maarten or (Trinidad adj2 Tobago) or (Turks adj2 Caicos Islands) or Virgin Islands or Uruguay or ((St Kitts or Saint Kitts) adj2 Nevis)).ti,ab,cp. (398497)

36 west indies/ or "antigua and barbuda"/ or bahamas/ or barbados/ or british virgin islands/ or cuba/ or dominica/ or dominican republic/ or grenada/ or guadeloupe/ or haiti/ or jamaica/ or martinique/ or netherlands antilles/ or puerto rico/ or "saint kitts and nevis"/ or saint lucia/ or "saint vincent and the grenadines"/ or "trinidad and tobago"/ or united states virgin islands/ or belize/ or costa rica/ or el salvador/ or guatemala/ or honduras/ or nicaragua/ or panama/ or panama canal zone/ or argentina/ or bolivia/ or brazil/ or chile/ or colombia/ or ecuador/ or guyana/ or paraguay/ or peru/ or uruguay/ or venezuela/ (157274)

37 caribbean region/ or central america/ or latin america/ or south america/ (27052)

38 (caribbean or west indies or central america or latin america or south america).ti,ab. (34075)

39 35 or 36 or 37 or 38 (456082)

40 4 and 39 (9400)

41 5 and 31 and 39 (12855)

42 40 or 41 (20770)

43 42 not 34 (19535)

44 limit 43 to (yr="1980 -Current" and (chinese or english or french or portuguese or spanish)) (18281)

45 limit 44 to exclude medline journals **(2279)**
